# Do assisted living facilities that offer a dementia care program differ from those that do not? A population-level cross-sectional study in Ontario, Canada

**DOI:** 10.1186/s12877-021-02400-w

**Published:** 2021-08-16

**Authors:** Derek R. Manis, Ahmad Rahim, Jeffrey W. Poss, Iwona A. Bielska, Susan E. Bronskill, Jean-Éric Tarride, Julia Abelson, Andrew P. Costa

**Affiliations:** 1 Centre for Health Economics and Policy Analysis, McMaster University, 1280 Main St W, CRL-201, Hamilton, ON L8S 4K1 Canada; 2 Department of Health Research Methods, Evidence, and Impact, McMaster University, 1280 Main St W, CRL-201, Hamilton, ON L8S 4K1 Canada; 3 ICES, Toronto, Ontario Canada; 4 School of Public Health and Health Systems, University of Waterloo, Waterloo, Ontario Canada; 5 Institute of Health Policy, Management & Evaluation, University of Toronto, Toronto, Ontario Canada; 6 Women’s College Research Institute, Toronto, Ontario Canada; 7 Sunnybrook Research Institute, Toronto, Ontario Canada; 8 Centre for Integrated Care, St. Joseph’s Health System, Hamilton, Ontario Canada; 9 Schlegel Research Institute for Aging, Waterloo, Ontario Canada; 10 Department of Medicine, McMaster University, Hamilton, Ontario Canada

**Keywords:** Assisted living facilities, Retirement homes, Dementia care, Canada

## Abstract

**Background:**

Many residents of assisted living facilities live with dementia, but little is known about the characteristics of assisted living facilities that provide specialized care for older adults who live with dementia. In this study, we identify the characteristics of assisted living facilities that offer a dementia care program, compared to those that do not offer such a program.

**Methods:**

We conducted a population-level cross-sectional study on all licensed assisted living facilities in Ontario, Canada in 2018 (*n* = 738). Facility-level characteristics (e.g., resident and suite capacities, etc.) and the provision of the other 12 provincially regulated care services (e.g., pharmacist and medical services, skin and wound care, etc.) attributed to assisted living facilities were examined. Multivariable Poisson regression with robust standard errors was used to model the characteristics of assisted living facilities associated with the provision of a dementia care program.

**Results:**

There were 123 assisted living facilities that offered a dementia care program (16.7% versus 83.3% no dementia care). Nearly half of these facilities had a resident capacity exceeding 140 older adults (44.7% versus 21.6% no dementia care) and more than 115 suites (46.3% versus 20.8% no dementia care). All assisted living facilities that offered a dementia care program also offered nursing services, meals, assistance with bathing and hygiene, and administered medications. After adjustment for facility characteristics and other provincially regulated care services, the prevalence of a dementia care program was nearly three times greater in assisted living facilities that offered assistance with feeding (Prevalence Ratio [PR] 2.91, 95% Confidence Interval [CI] 1.98 to 4.29), and almost twice as great among assisted living facilities that offered medical services (PR 1.78, 95% CI 1.00 to 3.17), compared to those that did not.

**Conclusions:**

A dementia care program was more prevalent in assisted living facilities that housed many older adults, had many suites, and offered at least five of the other 12 regulated care services. Our findings deepen the understanding of specialized care for dementia in assisted living facilities.

**Supplementary Information:**

The online version contains supplementary material available at 10.1186/s12877-021-02400-w.

## Introduction

Dementia affects more than half of all residents who reside in assisted living facilities [1–3]. Older adults who live with dementia are more likely to experience injuries requiring acute care, be diagnosed with pneumonia, and encounter difficulties with eating [4, 5]. Care for dementia is expensive and a widely cited reason for older adults requiring placement in a nursing home [6–9]. Specialized care for older adults who live with dementia, such as a dementia care program, has demonstrated reductions in acute health service use and transitions to a nursing home [8, 10].

Assisted living facilities provide congregate care in a residential setting to support independent living [11, 12], and assisted living facilities are referred to as retirement homes in Ontario, Canada. Assisted living facilities and retirement homes in the United States and Canada are regulated at the state- or provincial-level [12–14]. Ontario is the only jurisdiction that regulates the sector through an independent, not-for-profit regulator (i.e., Retirement Homes Regulatory Authority [RHRA]) [15]. All licensed assisted living facilities in Ontario must provide, at a minimum, any two of the 13 provincially regulated care services to six or more older adults [16]. The assisted living and retirement home sector in Ontario has a resident capacity equivalent to that of its nursing home sector (i.e., more than 70,000 older adults) [15], yet population-level studies of the sector pales in comparison to the nursing home sector. Unlike nursing homes, residency in a retirement home is exclusively financed through private, out-of-pocket payments by residents and/or their family caregivers [15, 17, 18].

Much of the literature on dementia care in assisted living facilities addresses health service use among residents who live with dementia, managing staff, and state-level regulations for dementia care [1, 14, 19–21]. Studies that explicitly investigate the characteristics of assisted living facilities or retirement homes that provide specialized care for dementia (i.e., a dementia care program), and how these characteristics compare to those that do not, has not received sufficient attention. The findings from such studies are important for identifying case mix and examining scope and breadth of care for older adults with complex care needs. A growing proportion of residents of assisted living facilities live with dementia [22], and improving the understanding of dementia care programs in assisted living facilities contributes to informing the sector, community-based dementia care, and national dementia care strategies.

In this study, we identify the characteristics of licensed assisted living facilities that offer a dementia care program compared to assisted living facilities that do not offer such a program in Ontario, Canada. As a dementia care program is the least prevalent regulated care service offered in assisted living facilities in Ontario, we hypothesize assisted living facilities that offer a dementia care program have an array of care services to support aging in place among residents. Our hypothesis is supported by other studies that investigated enhanced programming and special care units for dementia in assisted living facilities [23, 24].

## Methods

### Study design and setting

We conducted a population-level cross-sectional study in Ontario, Canada at ICES. ICES is an independent, non-profit research institute funded by an annual grant from the Ontario Ministry of Health (MOH) and the Ministry of Long-Term Care (MLTC). As a prescribed entity under Ontario’s privacy legislation, ICES is authorized to collect and use health care data for the purposes of health system analysis, evaluation, and decision support. Secure access to these data is governed by policies and procedures that are approved by the Information and Privacy Commissioner of Ontario. The REporting of studies Conducted using Observational Routinely-collected health Data (RECORD) statement guideline was followed (Supplemental Table S2) [25].

### Data and study population

A list of all licensed assisted living facilities in Ontario in 2018 was obtained from the public register of the RHRA and imported to ICES (*n* = 757). The postal code of each assisted living facility was linked to Statistics Canada’s Postal Code Conversion file, which is a specialized macro for use with health system administrative datasets containing postal codes. This macro is based on 2016 Census information, flags communities with a population less than 10,000 individuals as rural, and includes related data from Canada Post Corporation [26]. These datasets were linked using unique encoded identifiers and analyzed at ICES. Nineteen assisted living facilities (*n* = 19) were removed from the analysis because of missing facility-level and postal code data.

### Exposures

The exposures of interest are facility-level characteristics (i.e., urban location, resident capacity, total suites, chain facility, residential home status, and co-location with a nursing home) and the other 12 provincially regulated care services offered in an assisted living facility (i.e., assistance with bathing, hygiene, ambulation, feeding, and dressing; continence care; skin and wound care; provision of meals; administration of medications; pharmacist, nursing, and medical services) (Supplemental Table S1).

### Outcome

The primary outcome is whether the assisted living facility offered a dementia care program. Dementia care programs in assisted living facilities in Ontario are regulated to include communication strategies, mental stimulation activities, health and wellness monitoring and promotion, and identification of triggers for responsive behaviours [27]. These programs must also be supervised by a regulated health care professional (e.g., registered nurse, physician, etc.), align with current evidence and best practices for dementia care, and be evaluated annually [27].

### Statistical analysis

Counts, percentages, and standardized differences were calculated to describe the facility-level and care service characteristics of assisted living facilities that offered, and did not offer, a dementia care program. Multivariable Poisson regression with robust standard errors was used to model unadjusted and adjusted estimates with 95% confidence intervals to identify the characteristics of assisted living facilities associated with the provision of a dementia care program [28]. Tests were two-tailed, and the level of statistical significance was set at α = 0.05. The deviance goodness-of-fit test was calculated to assess whether the Poisson regression model was appropriate. Variance inflation factors were calculated to assess for multicollinearity. Dataset processing was conducted in SAS Enterprise 9.4 (Cary, NC, USA) and statistical analyses were conducted in Stata MP 16.1 (College Station, TX, USA).

## Results

There were 738 licensed assisted living facilities in Ontario in 2018 (*n* = 738). Of these, 123 offered a dementia care program (16.7% versus 83.3% no dementia care program), and almost all were located in an urban area (92.7% versus 82.6% no dementia care program) (Table 1). Nearly half of these assisted living facilities had a resident capacity of 140 or more (44.7% versus 21.6% no dementia care program) and had more than 115 suites (46.3% versus 20.8% no dementia care program). All assisted living facilities that offered a dementia care program also provided nursing services, meals, assistance with bathing and hygiene, and administered medications (*n* = 123). In addition, very few (i.e., six or fewer) assisted living facilities that offered a dementia care program did not offer assistance with ambulation and dressing, pharmacist services, and continence care. Many of the standardized differences between assisted living facilities that offered a dementia care program and those that did not exceeded 10%, which indicated that assisted living facilities that offered a dementia care program were systematically different from those that did not.

**Table 1:** Descriptive Characteristics of Licensed Assisted Living Facilities in 2018 (*n* = 738)

	Dementia Care Program		Standardized Difference
	Yes	No	
*n* (%)	123 (16.7)	615 (83.3)	
**Facility Characteristics, *n *(%)**			
Urban Location	114 (92.7)	508 (82.6)	0.309
Facility Capacity			0.583
6 to 49	24 (19.5)	155 (25.2)	
50 to 86	14 (11.4)	171 (27.8)	
87 to 139	30 (24.4)	156 (25.4)	
140+	55 (44.7)	133 (21.6)	
Total Suites			0.615
6 to 41	20 (16.3)	163 (26.5)	
42 to 70	16 (13.0)	168 (27.3)	
71 to 114	30 (24.4)	156 (25.4)	
115+	57 (46.3)	128 (20.8)	
Chain Facility	74 (60.2)	281 (45.7)	0.293
Residential Home	8 (6.5)	71 (11.5)	0.176
Co-Located with Nursing Home	19 (15.4)	112 (18.2)	0.073
**Care Services, *n *(%)**			
Assistance with Bathing	123 (100.0)	581 (94.5)	0.342
Assistance with Hygiene	123 (100.0)	531 (86.3)	0.562
Assistance with Ambulation	117 to 123 (95.1 to 100.0) ^a^	517 (84.1)	0.480
Assistance with Feeding	89 (72.4)	185 (30.1)	0.933
Assistance with Dressing	117 to 123 (95.1 to 100.0) ^a^	532 (86.5)	0.507
Continence Care	117 to 123 (95.1 to 100.0) ^a^	457 (74.3)	0.788
Skin and Wound Care	47 (38.2)	113 (18.4)	0.451
Provision of Meals	123 (100.0)	609 to 615 (99.0 to 100.0) ^a^	0.057
Administration of Medications	123 (100.0)	609 to 615 (99.0 to 100.0) ^a^	0.114
Pharmacist Services	117 to 123 (95.1 to 100.0) ^a^	535 (87.0)	0.287
Nursing Services	123 (100.0)	574 (93.3)	0.377
Medical Services	107 (87.0)	401 (65.2)	0.528

^a^Small cell sizes (i.e., where six or fewer assisted living facilities have, or do not have, a characteristic) are suppressed due to privacy restrictions at ICES.

Assistance with bathing and hygiene, provision of meals, administration of medications, and nursing services were removed from the adjusted model because of collinearity, and there was no evidence of multicollinearity in the adjusted model (i.e., variance inflation factors equal to or greater than a value of 10). The deviance goodness-of-fit statistic was not statistically significant. After adjustment for facility characteristics and regulated care services, the prevalence of a dementia care program was almost three times greater in assisted living facilities with 115 or more suites (Prevalence Ratio [PR] 2.78, 95% Confidence Interval [CI] 1.09 to 7.07) compared to assisted living facilities with 41 or fewer suites (Table 2). The prevalence of a dementia care program was nearly three times greater in assisted living facilities that offered assistance with feeding (PR 2.91, 95% CI 1.98 to 4.29), and the prevalence of a dementia care program was almost twice as great in assisted living facilities that offered medical services (PR 1.78, 95% CI 1.00 to 3.17), compared to assisted living facilities that did not offer these care services. The prevalence of a dementia care program was substantially greater in assisted living facilities that offered continence care (PR 13.51, 95% CI 1.64 to 111.67) compared to assisted living facilities that did not offer this care service.

**Table 2:** Associations with the Provision of a Dementia Care Program in Licensed Assisted Living Facilities

	Unadjusted PR (95% CI)	Adjusted PR (95% CI)^a^
**Facility Characteristics**		
Urban	2.26 (1.23 to 4.52) ^**^	1.15 (0.61 to 2.17)
Facility Capacity		
6 to 49	1.00 (Reference)	1.00 (Reference)
50 to 86	0.56 (0.30 to 1.06)	0.34 (0.18 to 0.66) ^**^
87 to 139	1.20 (0.73 to 1.98)	0.43 (0.20 to 0.93) ^*^
140+	2.18 (1.41 to 3.37) ^***^	0.59 (0.25 to 1.42)
Total Suites		
6 to 41	1.00 (Reference)	1.00 (Reference)
42 to 70	0.80 (0.43 to 1.49)	1.40 (0.73 to 2.70)
71 to 114	1.48 (0.87 to 2.50)	2.28 (1.02 to 5.11) ^*^
115+	2.82 (1.77 to 4.50) ^***^	2.78 (1.09 to 7.07) ^*^
Chain Facility	1.63 (1.17 to 2.27) ^**^	1.21 (0.88 to 1.67)
Residential Home	0.58 (0.29 to 1.14)	0.75 (0.35 to 1.61)
Co-Located with a Nursing Home	0.85 (0.54 to 1.33)	1.21 (0.78 to 1.87)
**Care Services**		
Assistance with Ambulation	6.34 (2.05 to 19.57) ^**^	0.96 (0.34 to 2.75)
Assistance with Feeding	4.43 (3.07 to 6.39) ^***^	2.91 (1.98 to 4.29) ^***^
Assistance with Dressing	15.67 (2.22 to 110.82) ^**^	2.24 (0.26 to 18.96)
Continence Care	33.50 (4.71 to 238.20) ^***^	13.51 (1.64 to 111.67) ^*^
Skin and Wound Care	2.23 (1.62 to 3.07) ^***^	1.18 (0.85 to 1.63)
Pharmacist Services	2.57 (1.17 to 5.66) ^*^	0.91 (0.38 to 2.21)
Medical Services	3.03 (1.83 to 5.00) ^***^	1.78 (1.00 to 3.17) ^*^

*Abbreviations*: *PR* Prevalence Ratio, *CI* Confidence Interval

^a^Adjusted for all variables in the table

^*^P < .05; ^**^P < .01; ^***^P < .001

## Discussion

Assisted living facilities that offered a dementia care program were systematically different from those that did not offer such a program. Specifically, assisted living facilities in Ontario that offered a dementia program had large resident capacities, many suites, and offered, at a minimum, nursing services, meals, assistance with bathing and hygiene, and administered medications. The prevalence of a dementia care program in an assisted living facility was greater in assisted living facilities where assistance with feeding, medical services, and continence care were also offered.

More than 90% of assisted living facilities that offered a dementia care program were located in urban communities. Consistent with existing literature, this finding raises important equity considerations for older adults who live with dementia in assisted living facilities located in rural and remote regions [29]. Rural assisted living facilities house fewer older adults and are more likely to have deficiencies in care provision than urban ones, including challenges with retaining appropriate care staff and resources to meet the needs of residents [30]. The use of videoconferencing and other information technology resources to offer dementia care should be considered to improve access to care for older adults who live with dementia in rural and remote areas [31].

Most assisted living facilities that offered a dementia care program had capacity for more than 140 older adults and had more than 115 suites. Current practices for designing settings specifically for older adults who live with dementia emphasize larger spaces that are not characteristic of institutionalized congregate care [32], and the presence and statistically significant association of many suites in assisted living facilities that offer a dementia care program aligns with the literature. In addition, this may indicate that many assisted living facilities that offer a dementia care program are large complexes, likely attributed to chains.

Given the challenges that older adults who live with dementia face with respect to eating [5], it is expected that assistance with feeding would be a prevalent care service offered alongside a dementia care program in an assisted living facility. Moreover, the complex and intersecting care needs of older adults who live with dementia, which includes polypharmacy [33], underscores the need for on-going medical care. As such, the prevalence of medical services in assisted living facilities that offer a dementia care program is also expected. There was a greater proportion of assisted living facilities that offered skin and wound care among assisted living facilities that offered a dementia care program compared to those that did not. However, there was no statistically significant association with this care service and the provision of a dementia care program in the adjusted model. This finding raises important safety considerations, as residents of assisted living facilities who live with advanced dementia may be bed-bound or have mobility issues that can contribute to the development of pressure ulcers [34].

As the assisted living sector is privately financed in Ontario, our study makes an important contribution to the literature to define the sector by modeling facility-level characteristics associated with the provision of a dementia care program. Our findings are relevant to clinicians and policymakers actively considering dementia care options in communities to support older adults who live with dementia and their caregivers. Family caregivers and consumers of assisted living services will also be interested in our findings to inform their decisions for housing and congregate care.

In North America, the regulatory requirements for assisted living facilities vary between all states and provinces [34]. In all other provinces and territories in Canada, assisted living facilities are periodically inspected by the government for compliance with the legislative and regulatory requirements in their jurisdiction. The assisted living sector has substantially grown over the past decade in response to the varying health and social needs and preferences of older adults for care and housing [11, 17, 18, 34]. Understanding the characteristics of assisted living facilities that offer a dementia care program informs national dementia care strategies to support older adults to age in place and reduce the demand for a bed in a nursing home associated with advanced dementia [35].

With respect to limitations, the fees charged by assisted living facilities for room and board and care services each month could not be included in the adjusted model. This is due, in part, to the inability to retrieve this information from existing administrative health system data. Moreover, these fees are not publicly available on the websites of assisted living facilities, through their member associations, or available to the RHRA through regulatory reporting requirements. In addition, variables related to staff training, type, and ratios were unavailable, as there are no regulatory reporting requirements of these to the RHRA as a condition for licensing. Another limitation is that our study is descriptive; as such, no causal or temporal claims can be made about the associations between the facility-level characteristics of assisted living facilities and the provision of a dementia care program. As with all secondary analyses of data, the data used in our study are susceptible to misclassification bias.

## Conclusions

Our study identified and compared facility-level characteristics of licensed assisted living facilities that offered a dementia care program to those that did not in Ontario, Canada in 2018. Assisted living facilities that offered a dementia care program housed more older adults and provided more care services. Future research might consider investigating the underlying differences in populations between residents of these facilities and their health outcomes attributed to care services offered in assisted living facilities. In addition, research that examines the quality of dementia care programs and the attributes of these programs is warranted.

## Supplementary Information

**Additional file 1: Supplemental Table S1.** Detailed Descriptions of Exposures. **Supplemental Table S2.** RECORD Checklist.

## Data Availability

The dataset from this study is held securely in coded form at ICES. While legal data sharing agreements between ICES and data providers (e.g., healthcare organizations and government) prohibit ICES from making the dataset publicly available, access may be granted to those who meet pre-specified criteria for confidential access, available at (email: das@ices.on.ca). The full dataset creation plan and underlying analytic code are available from the authors upon request, understanding that the computer programs may rely upon coding templates or macros that are unique to ICES and are therefore either inaccessible or may require modification.

## Abbreviations

CI: Confidence interval
MLTC: Ontario Ministry of Long-Term Care
MOH: Ontario Ministry of Health
PHIPA: Ontario Personal Health Information and Protection Act
PR: Prevalence Ratio
RECORD: Reporting of studies Conducted using Observational Routinely-collected health Data
RHRA: Retirement Homes Regulatory Authority
